# Ancient inhabitants of the Basin of Mexico kept an accurate agricultural calendar using sunrise observatories and mountain alignments

**DOI:** 10.1073/pnas.2215615119

**Published:** 2022-12-12

**Authors:** Exequiel Ezcurra, Paula Ezcurra, Ben Meissner

**Affiliations:** ^a^Department of Botany and Plant Sciences, University of California Riverside, Riverside, CA 92521-0147; ^b^Climate Science Alliance, San Diego, CA 92169; ^c^Independent Filmmaker/Photographer, Sioux Falls, SD 57104

**Keywords:** Basin of Mexico, Mesoamerican calendar, pre-Hispanic farming, Mount Tlaloc

## Abstract

In the hot dry spring of monsoon-driven environments, keeping an accurate calendar to regulate the annual planting of crops is of critical importance. Before the Spanish conquest, the Basin of Mexico had a highly productive farming system able to feed its very large population. However, how they managed to keep their farming dates in synchrony with the solar year is not known. In this paper, we show that the observation of sunrise against the Basin’s eastern horizon could have provided an accurate solar calendar and that some important sunrise landmarks coincide well with the themes of seasonal festivities described in early codices. We also show that a long stone causeway in the summit of Mount Tlaloc aligns perfectly with the rising sun on February 23 to 24, in coincidence with the Basin’s new year in the Mexica calendar. Third, we demonstrate that, when viewed from the sacred Mount Tepeyac in the bottom of the Basin, sunrise aligns with Mount Tlaloc also on February 24. The importance of Mount Tlaloc as a calendric landmark seems to be corroborated by illustrations and texts in ancient Mexica codices. Our findings demonstrate that by using carefully developed alignments with the rugged eastern horizon, the inhabitants of the Basin of Mexico were able to adjust their calendar to keep in synchrony with the solar year and successfully plan their corn harvests.

## Significance

Without the navigational and calendric instruments of the 16th century Europeans (like gnomon, compass, quadrant, or astrolabe), the inhabitants of the Basin of Mexico were able to keep an accurate agricultural calendar that allowed them to plan their agricultural cycle to feed one of the largest population densities on Earth, as well as maintaining rituals associated to the solar seasons. To achieve this, they used the rugged topography of the Basin as a precise solar observatory and also built a high-altitude stone causeway for accurate adjustments of their calendar to the solar year. These results underscore how a similar goal, such as adjusting the length of the calendar to the solar year, could be achieved with widely different technologies.

**Fig. 1.**
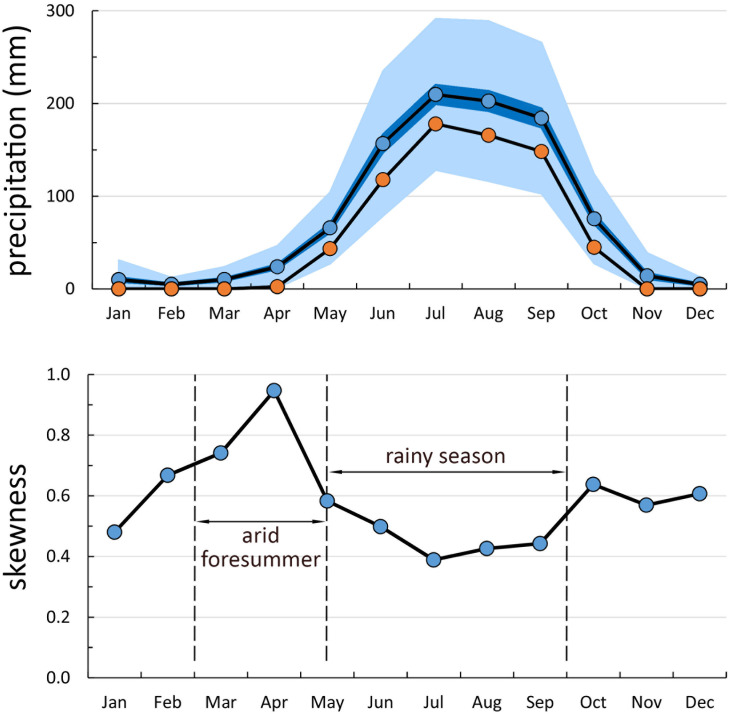
(*A*) Precipitation pattern in the Basin of Mexico. Blue points: monthly means; orange points: monthly modes; blue shaded area: ±1 SE; light blue area: ±1 SD. (*B*) Pearson skewness, a measure of risk from rare extreme events. Note the high skewness of the dry spring months, March to mid-May (data from years 1952 to 2016, Weather station 9020 Pedregal, *Comisión Nacional del Agua*, Mexico).

In 1519, at the time of the arrival of the Spanish invaders to the Basin of Mexico, the people in the region ran a sophisticated system of agriculture that was able to feed its large human population, estimated by different studies between 1 and 3 million (1). Successful farming in central and western Mesoamerica depended critically on the ability to keep an accurate calendar to predict the seasons. Apart from the wet tropics of the coastal plains of the Gulf of Mexico and the Caribbean, all other regions of Mesoamerica, namely the Mexican Altiplano, the Balsas Basin, and the seasonally dry ecosystems of the Pacific slopes of Mexico, share a highly cyclical precipitation pattern with a dry spring followed by a monsoon-type rainy season in summer and early fall. Precipitation-wise, the most unpredictable time of the year is mid-, and in some parts late, spring; the “arid fore-summer” that precedes the arrival of the Mexican monsoon (Fig. 1 and *SI Appendix*, Fig. S1). Planting too early, following the cue of a first haphazard early rain, can be disastrous if the true rainy season does not continue. Waiting to plant late, after the monsoon season has clearly started, can expose the corn field, or *milpa*, to an overly short growing season and will also put the crop under competition from weeds that have already germinated. Wild plants in these highly seasonal ecosystems often have traits that allow them to hedge the risk of a false moisture cue. Annual plants often have heteromorphic seeds, some of which germinate with a single rain pulse while others remain dormant and germinate after successive rainfall events (2). Other plants have lignified seed-retention structures that release seeds gradually into the environment as the dry spring progresses (3). Finally, woody perennials often flower in the dry early spring in response to photoperiod, independently of moisture availability, and shed their seeds in late spring or early summer (May–June) when the monsoon season is starting (4). In this latter group, the physiological ability to detect the season independently of precipitation cues is critically important to avoid premature germination. Accurate timekeeping must have also been strategically critical for pre-Hispanic farmers, who, in order to be successful, had to prepare the *milpa* fields before the onset of the monsoon rains and plant as early as possible while, at the same time, being able to disregard false early rainfall signals. In the 16th century, Diego Durán (5) noted the importance that the native calendar had for these communities “to know the days in which they had to sow and harvest, till, and cultivate the corn field.” He also noted as “a very remarkable fact” that the Mexica farmers followed strictly the calendrically based instruction of the elders to plant and harvest their fields and would not start their farming activities without their approval.

Because of the calendar’s importance for societal organization, the early chroniclers of Aztec, or Mexica, civilization, in particular, Bernardino de Sahagún (6) and Diego Durán (5), left behind detailed descriptions of their calendar system. They both underscored the precision of the method used to keep track of seasons and the fact that the Mexica were well aware of the need to adjust their calendar by adding an extra day every four years to the annual count in order to keep the march of the seasons in tune with their calendrical computation. However, other early chroniclers such as Motolinía (7), as well as modern researchers (8–10), have doubted that the Mexica could have had a leap-year count system, and the debate continues to this day. Nevertheless, many modern researchers that doubt the existence of a systematic leap-year count concede that the Mexica calendar was not out of phase with seasonal change along the solar year and that some nonsystematic mechanism must have existed for adjusting the calendar system at irregular intervals (11). The renowned Mexican historian Rafael Tena (12) concluded that “this fascinating problem remains unresolved and open to discussion.”

In order to adjust their calendar, the Mexica would have needed to know the position of the sun on particular dates of the solar year, a feat that could have been accomplished only by marking the sunrise (or sunset) bearing relative to a geographic landmark. Many studies have analyzed the alignment of temples and ceremonial centers with the sun’s azimuthal bearing at sunrise or sunset on culturally relevant dates, so the architectural orientation of major buildings such as the Templo Mayor, and in general the urban design, would reflect important calendar dates (11). This architectural design would have had a great symbolic, ritual, and cultural importance but would have not been the most accurate way of measuring the annual march of time because of parallax error: small shifts in the position of the observer relative to a building or ceremonial structure can project large errors in the distant horizon.

Because Mesoamerican settlements were all located inside the tropics, i.e., south of latitude 23.5°N, the zenithal transition of the sun occurs here twice every year; first, in late May as the sun’s trajectory in the celestial sphere moves northward toward the summer solstice, and then back in late July, as the sun’s trajectory returns southward (the exact date of the zenithal transition depends on the latitude of the site). Two settlements south of the Basin of Mexico—Xochicalco and Monte Albán—had specially constructed “zenith tubes;” vertical shafts perforated on the rock or constructed inside large ceremonial buildings that would project direct sunlight onto an observation chamber belowground (13, 14). The projection of these solar flecks on the ground would have allowed observers to keep track of exact solar dates and, potentially, to use them for calendric adjustments (15–17). However, no evidence exists that zenith tubes were ever used in the Basin of Mexico, and many scholars believe that the calendar-keepers of Tenochtitlan directly used the sun’s position at sunrise against the prominent peaks on the basin’s mountainous horizon as calendrical landmarks, a “horizon calendar” that provided accurate indicators of specific dates along the solar year (18, 19).

The use of the landscape as a calendric tool is based on the fact that because of the tilting of the Earth, the point in the horizon from where the sun rises shifts day-to-day along the year (18). In reality, the sun rises due east only during the equinoxes (near March 21 and September 22). In the Northern Hemisphere summer when the North Pole is tilted toward the sun, the sun in the Basin of Mexico will rise north of due east in the celestial horizon, reaching a compass bearing of ca. 65° during the summer solstice. Likewise, in winter, it will rise south of the 90° east bearing, reaching an azimuth of ca. 115° during the winter solstice. The azimuth angle of the sun on the celestial horizon at sunrise is a function of the declination of the Sun from the celestial equator at any given date and the latitude of the observation point (20). If the observer is on a fixed location, say, the Templo Mayor, then latitude is constant and the solar azimuth at sunrise becomes a direct function of the winter-to-summer declination of the sun, which is in turn a function of the date in the solar year. So, by looking at sunrise against a distant mountainous landscape from a fixed point, an observer can keep tab on the days in a year with minimal parallax error.

Landscape features are still often used to mark calendric dates in many traditional villages. Despite modern communications and the standardization of calendric time, many communities still have ceremonies related to planting or harvest that are celebrated when sunrise or sunset is aligned with mountains that act as reference points (21). Using landscape silhouettes in the horizon as a means for keeping count of time and seasons has been so important in the past that some cultures have even built their own calendric reference points to use as a sunrise observatory in flat terrains, like the towers of Chankillo in north coastal Peru, built in the 4th century BCE (22). In the Basin of Mexico, as in many other parts of the Americas, the alignment of the rising sun with mountains seems to have been a common calendric and navigational tool. For example, the *Florentine Codex* (6) (book 11, Eighth Chapter) describes how mineral experts used the alignment of sunrise against surrounding mountains to mark and relocate mineral deposits (*SI Appendix*, Fig. S2).

## Goals and Hypotheses

The central hypothesis of this study is that the eastern mountain landscape of the Basin of Mexico played a central role as a tool for adjusting the calendar system to the solar year. Many studies have explored the symbolic and ritual importance of mountains as calendric tools. In contrast, we will concentrate on the ecological and agricultural importance of the Basin’s landscape as a tool for accurate calendric adjustments. Our study concentrates on the *xiuhpohualli* or “year count,” the solar calendar that regulated the cycles of agriculture (*SI Appendix*, endnote 2). We explore four hypotheses: a) The eastern horizon landmarks could have provided a series of reference points that would have allowed the adjustment of the agricultural calendar to the solar year. b) There is evidence in the texts of early chroniclers and codices that the Mexica people were indeed using horizon landmarks to follow the dates of the solar year. c) An ancient construction in the summit of Mount Tlaloc seems to have been used as a fixed solar observatory built for the purpose of calendric adjustments. d) Before the foundation of Tenochtitlan and the establishment of the Aztec dominion in the Basin of Mexico, other large agricultural civilizations that preceded them were also using the Basin’s mountainous horizon for calendric purposes.

## Results

### Sunrise Alignments.

**Fig. 2.**
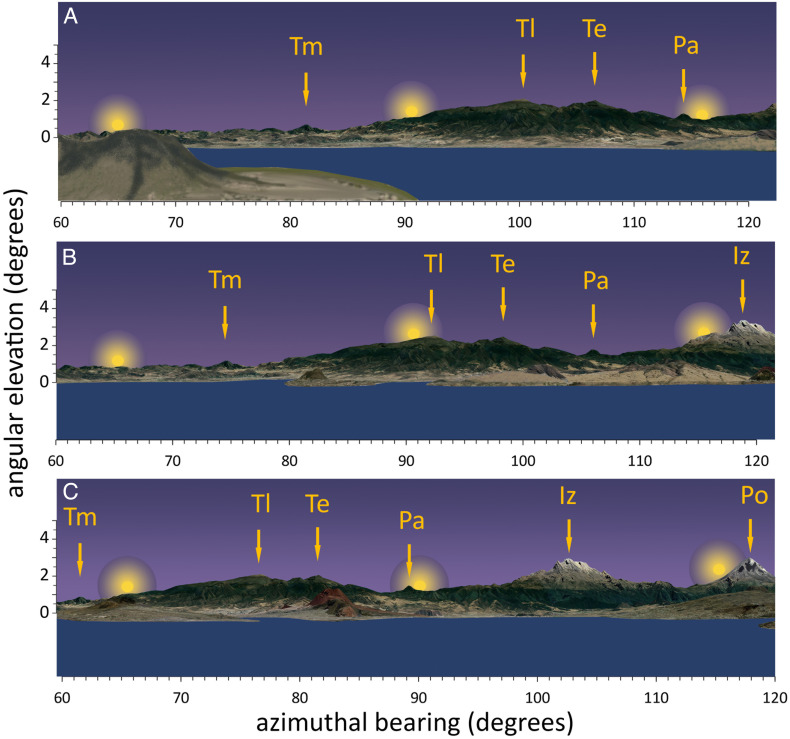
The eastern horizon of the Basin of Mexico, as viewed from (*A*) Mount Tepeyac, (*B*) Templo Mayor, and (*C*) the pyramid of Cuicuilco. From NE to SE, labels highlight all major horizontal landmarks: Tlamacas (Tm), Mount Tlaloc (Tl), Telapon (Te), Papayo (Pa), Iztaccihuatl (Iz), and Popocatepetl (Po). The simulated solar disks indicate the position of the sun at dawn during the summer solstice (*Left*), equinox (*Center*), and winter solstice (*Right*). Horizon images were obtained using Google Earth©.

The mountainous landscape east of the Basin of Mexico offers some important topographic markers that could have been used by Mexica astronomers for calendrical purposes. For example, viewed from the top of the Templo Mayor, sunrise during the winter solstice would have broken at an azimuth of 116°22′ and an angular elevation of 3°20′ behind Mount Tehuicocone, a prominence on the northern slope the great Iztaccíhuatl volcano and very close to the “head” of the volcano’s “sleeping woman” silhouette (Table 1 and Fig. 2*B*). During the summer solstice, the sun would have been seen rising behind the settlement of Tepetlaoxtoc, in the foothills of the low Sierra de Patlachique, north-east of Texcoco, with an azimuth of 65°04′ and an angular elevation of 10′. On March 16 and September 30, the sun would have been seen rising behind the peak of Mount Tlaloc, close to due east, with an azimuth of 93°17′ and an angular elevation of 2°51′. Finally, on March 1 and October 15, the sun would have been seen rising behind the peak of Mount Telapón with an azimuth of 98°55′ and an angular elevation of 2°45′ (all dates in this study follow the Gregorian calendar established in 1582, not the Julian Calendar used by early 16th century scholars and chroniclers, which differed at that time by 10 d).

**Table 1.** Sunrise alignment dates (Gregorian calendar days) between the three main astronomical observation sites in the Basin of Mexico and seven conspicuous landmarks on the Basin’s eastern horizon

	Observatories		
Horizon landmarks	Tepeyac	Templo Mayor	Cuicuilco
	Spring semester (Jan–June)		
Tlamacas	12-Apr	1-May	–
Monte Tlaloc	24-Feb	16-Mar	28-Apr
Telapon	7-Feb	1-Mar	14-Apr
Papayo	7-Jan	6-Feb	24-Mar
Iztaccihuatl (head)	–	–	20-Feb
Iztaccihuatl (peak)	–	–	18-Feb
Popocatepetl	–	–	–
	Fall semester (July–December)		
Tlamacas	2-Sep	14-Aug	–
Monte Tlaloc	19-Oct	30-Sep	17-Aug
Telapon	5-Nov	15-Oct	31-Aug
Papayo	7-Dec	6-Nov	21-Sep
Iztaccihuatl (head)	–	–	23-Oct
Iztaccihuatl (peak)	–	–	25-Oct
Popocatepetl	–	–	–

Not all these landmark points, however, have the same resolution for the purpose of keeping count of the days in the solar year. The solar azimuth at sunrise will vary at the latitude of the Basin of Mexico from summer to winter forming a wave-like function along the year. The changes in azimuth from one day to the next—i.e., the azimuthal shifts, or difference between the sunrise azimuth one day and that of the previous day—form a similarly shaped function displaced ca. 91 d (*SI Appendix*, Fig. S3). During the spring equinox the daily azimuthal shift at sunrise will be −0.418° (−25′ d^−1^). During the fall equinox the azimuthal shift will be numerically similar but opposite in sign (25′ d^−1^). Near the summer and winter solstices, sunrise will seem to stand still for some 10 d, shifting its sunrise azimuth in 2′ or less per day and appearing to rise in the horizon from the same spot (hence the name *solsticium* in Latin). The angular size of the sun’s disk is ca. 31′, a value only slightly larger than the daily shift in sunrise azimuth during the equinox (25′). This means that an observer seeing the sun rise behind a horizon landmark near the equinox—like, for example, Mount Tlaloc—would see sunrise azimuths shift daily by a value that is 81% as large as the sun’s apparent diameter. In simple terms, there is only 1 d in the spring and 1 d in the fall in which the sun can be seen rising exactly behind Mount Tlaloc. In contrast, during the solstices, when the sun reaches its maximum declination, the sunrise azimuth varies in less than 2′, that is, one-fifteenth of the angular size of the solar disk, an amount that is not perceptible to the naked eye. For a Mexica astronomer following the calendric horizon, during late December and late June, the point of sunrise would seem to stand unchanging for at least 10 d.

It seems, then, reasonable to assume that Mexica astronomers keeping a record of the calendric horizon would have used Mount Tlaloc, the horizontal point of reference nearest to the equinox, as their fundamental tool for calendar counts and calendric adjustments, because this landmark would have given them maximum calendric precision. Following this approach, the number of days in a year could be counted from the day that the sun rises behind Mount Tlaloc in spring to the next springtime sunrise behind Mount Tlaloc. The count would be 365 d: the 18 Mexica “months” of 20 d each plus the 5 *nemontemi*, or “useless” days, at the end of the year. But anyone keeping count of days this way would observe a gradual displacement of the sun’s sunrise position because the true length of the solar year is closer to 365.2422 d. After four years, adding an extra *nemontemi* day to adjust the day count to the horizon calendar would have been necessary in order to keep the sun rising behind the peak of Mount Tlaloc for the same day of the year. This hypothesized adjustment using the distant horizon, however, is only possible, as discussed above, if a landmark point near the equinox is used for the calendric adjustments, so that the precise day of the year can be identified without error.

### Corroborating Evidence: The Florentine Codex.

According to Sahagún’s 1575 description (6), the new year in the Basin of Mexico started on February 2 of the Julian Calendar in use at that time, which translates to February 12 of our current Gregorian calendar. However, comparing dates of historical events between 1519 and 1521 that were recorded both in Mexica codices and in Spanish chronicles, Tena (23) estimated that at the time of the arrival of the first Europeans to Mexico, the first month of the Mexica calendar, *Atlcahualo*, began on February 13 of the Julian calendar or February 23 of the Gregorian calendar. In later papers (12, 24), Tena added three more days to compensate for the suppression of leap days in the secular years of 1700, 1800, and 1900, arguing that the Mexica calendar year started on February 26. Although his recent correction might be relevant to convert modern dates into the Mexica calendar, it seems clear that in the 16th Century, when the Gregorian calendar was established, the Mexica year started on February 23.

Following Tena’s chronography, viewed from the Templo Mayor sunrise aligned with Mount Tlaloc 5 d before the equinox, at the end of *Atlcahualo*. In his description of the Mexica calendar, Sahagún (6) noted that on *Atlcahualo* the Mexica “celebrated a feast […] to the Tlaloc gods, whom they held to be gods of rain.” The tributes to Tlaloc continued during the dry fore-summer: Sahagún also narrates that at the beginning of the third month—*Tozoztontli* ca. April 4—“a feast was made to a god called Tlaloc, who is the god of rains.” The clear association between Tlaloc, the equinox, and the dry springtime supports the assumption that sunrise behind Mount Tlaloc, viewed from Tenochtitlan, marked the highpoint of Mesoamerica’s premonsoon dry spring and led the people of the Basin to plead to Tlaloc for the arrival of the rains.

Similarly, according to Tena’s chronography, the summer solstice took place toward the end of the sixth month, called *Etzalqualiztli*. At that time, sunrise would have seemed to stand still at an azimuth of ca. 65°. Viewed from the top of the Templo Mayor, sunrise would have taken place behind Tepetlaoxtoc, in the western foothills of the Sierra de Patlachique, across the briny waters of Lake Texcoco where the Basin’s saltworks were (25–27). In coincidence, the first day of the seventh month, called *Tecuilhuitontli*, was devoted to a celebration in honor of *Huixtocihuatl*, the goddess of salt. Close to the summer solstice bearing, further east from the salty lakeshores, there were fertile agricultural terraces with cultivated *milpas*, or cornfields. Sahagún noted that in the eighth month, called *Huey Tecuilhuitl*, the goddess of fresh corn, *Xilonen*, or *Chicomecoatl*, was also celebrated. It does not seem coincidental that the name of Chiconcuac, a settlement found along this summer sunrise view, is derived from the name of this goddess.

The winter solstice occurred close to the beginning of the 16th month, *Atemoztli*, a time in which sunrise seems to stand still at its southernmost azimuth of ca. 116°, on the northern slope of the Iztaccíhuatl volcano, the “sleeping woman” (the name Iztaccíhuatl in Nahuatl means “white woman,” in allusion to the snow-covered silhouette as seen from the Templo Mayor). According to Sahagún, the beginning of the following month, called *Tititl*, was devoted to celebrating *Ilama Tecuhtli* (the Great Lady), also known as *Tona* (Our Mother). The correlation between sunrise close to the woman-like volcano and the celebration of womanhood in general is striking.

In summary, there seems to be a noteworthy association between some elements of the horizon calendar and the feasts and celebrations of each season: the arid spring equinox, when the sun rises behind Mount Tlaloc, was associated with Tlaloc, the god of water and rain. The summer solstice, when sunrise occurs behind the distant salty shores of Lake Texcoco, was associated with salt and summer corn. Finally, the winter equinox, when the sun rises at the side of Iztaccihuatl, the sleeping woman, was associated with womanhood and female gods.

### The Causeway of Mount Tlaloc.

The previous analysis suggests a correlation between the Mexica calendar and the topographic elements of the Basin’s eastern horizon but leaves an important question unanswered, namely that of the calendric role of Mount Tlaloc. It seems very clear that the horizon calendar, as viewed from Tenochtitlan’s Templo Mayor, should have relied strongly on the date of the sun rising behind Mount Tlaloc, as this mountain could have provided, better than any other, the accuracy needed for the precise estimation of the length of the solar year and for leap year adjustments. However, none of the 16th century codices and manuscripts consulted for this study describe this phenomenon in a direct and clear manner, other than a general mention in Sahagún that at the beginning of the third month, close to the alignment date of sunrise with Mount Tlaloc, a feast was made to Tlaloc, the god of rains. If the alignment of sunrise with Mount Tlaloc was indeed an important calendric landmark when viewed from Templo Mayor, a clear mention could have been expected in the ancient codices, including the question of why did the Mexica not use the Mount Tlaloc alignment to mark the beginning of the new year. The answer to this paradox may lie in the ruins of the ceremonial center found at Mount Tlaloc’s peak.

**Fig. 3.**
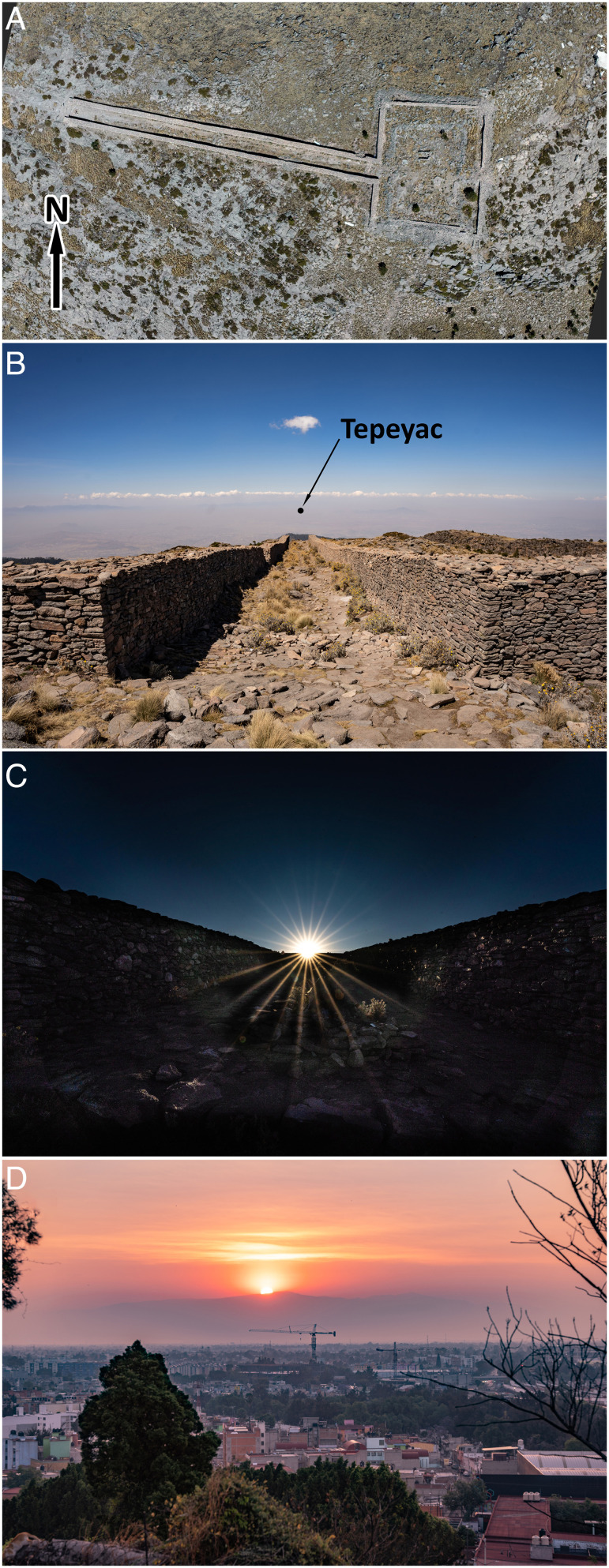
(*A*) The Mount Tlaloc square courtyard (*tetzacualo*) and stone causeway, photographed using a drone on February 24, 2022. (*B*) Downslope view of the causeway from the *tetzacualo* toward the Basin of Mexico, with Mount Tepeyac indicated (not clearly visible otherwise in the smog of Mexico City). (*C*) Upslope view of sunrise from the base of the causeway toward the *tetzacualo* on February 25, 2022, at 7:20 h Mexico City time (GMT-6). (*D*) Sunrise viewed from Mount Tepeyac on February 26, 2022, at 7:10 h. Note that, because the alignment date was 2 d earlier, the rising sun is displaced ca. 1° north of the peak of Mount Tlaloc, visible in the distance. Photo credits: Ben Fiscella Meissner.

**Fig. 4.**
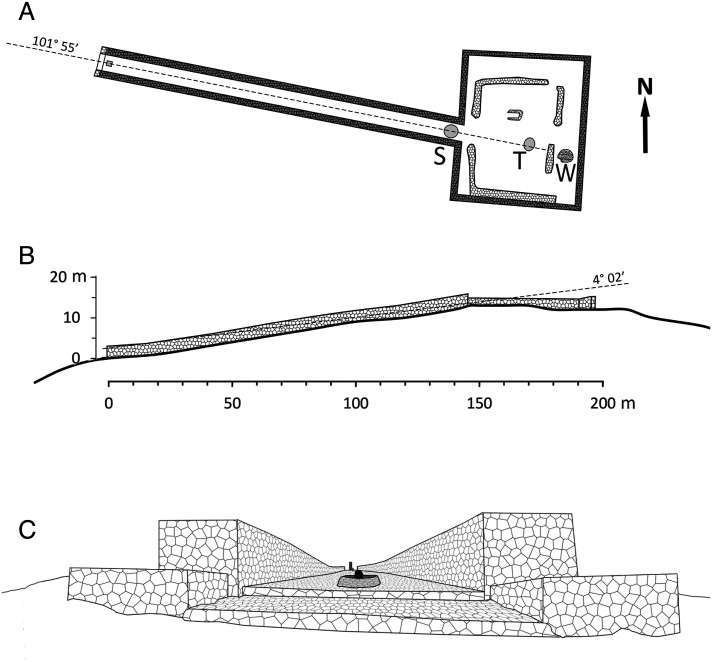
(*A*) The Mount Tlaloc square courtyard (*tetzacualo*) and stone causeway, vertical projection elaborated from a drone digital image. The stone circle in the upper causeway (S) and the rock platform where Tlaloc’s monolith stood (T), visible in the drone image, together with the dugout well (W), are marked for reference. (*B*) An elevation plan, or longitudinal section, along the main axis of the causeway, shows the angular elevation of the celestial bearing. (*C*) Upslope view along the causeway, showing the alignment of Tlaloc’s monolith in the courtyard with the center of the stone circle in the upper causeway and the existing stone marker in the entrance below.

The summit of Mount Tlaloc is crowned by a rectangular walled enclosure about 40 m east–west by 50 m north–south (Figs. 3*A* and 4). This courtyard, or *tetzacualo*, consists of stone walls that have been estimated to have been 2 to 3 m high when originally built, with a ca. 94° east-west azimuth (28, 29). The eastern side of the precinct opens to a 150 m-long, ca. 6 m-wide, walled straight causeway that has an azimuthal bearing of 101°55′, offset more than 8°southward from the roughly east–west bearing of the enclosure (Fig. 3*B*). Because the causeway runs downslope on the western side of the peak, some researchers have wondered whether the causeway was intentionally misaligned with the axis of the enclosure in order to accommodate a particular orientation to the setting sun (30, 31).

If viewed upslope, the azimuthal bearing of Mount Tlaloc’s causeway (101°55′) and the angular elevation of 4°02′ above the celestial horizon (allowing for the height of the viewer’s eyes) defines a point in the celestial sphere that aligns with the sun’s apparent position on February 23 to 24 each year. That is, an observer standing at the lower end of the causeway will see the rising sun appear in the center of the upper part of the stone ramp on February 23 or 24, after the last *nemontemi* day and in synchrony with the beginning of Basin’s new year as defined by Tena’s first chronology (Fig. 3*C* and *SI Appendix*, Figs. S4 and S5). The causeway seems to have been constructed as a calendric solar marker with a celestial bearing that allows for leap-year adjustments and indicates the end of the year and the beginning of a new solar year. The idea that the structure was used for precise astronomical observations is further reinforced by the fact that it seems to have had specific sight markers to avoid parallax error. Wicke and Horcasitas (32) described that the causeway had a stone circle in its upper end where, presumably, a monolith could have stood. Correspondingly, it still has a stone square with an erect, 40-cm monolith in its lower end. Jointly, they could have been used as alignment markers (similar to the iron sights of a gun) to further improve alignment accuracy. Almost a century ago, Rickards (33) described the presence of a monolith with the figure of Tlaloc in the center of the *tetzacualo* and aligned with the causeway, as had been described earlier by Durán (5). Although the figure has been removed since (31), it could have functioned as yet another element for precise solar alignments (Fig. 4).

The importance of Mount Tlaloc as a solar observatory is enhanced by the fact that the two largest peaks of the Mexican Transversal Volcanic Axis east of the Basin of Mexico are visible from its peak and almost perfectly aligned. Viewed from the center of the stone courtyard, the nearest peak, Matlalcuéyetl or Malinche (19°13.70′, −98°01.94′) has an azimuth of 105°52.7′ while Citlaltépetl or Pico de Orizaba (19°01.78′, −97°16.15′) has an azimuth 105°26.5′. Because the azimuthal difference between the two peaks is less than the angular width of the sun’s disk, viewed at dawn they will seem like a single mountain with two close crests, where sunrise would be seen on February 10. In short, the causeway in Mount Tlaloc marks very precisely the beginning of the Mexica solar year, but the summit courtyard could have been used to identify a precise alignment 15 d before the beginning of the year, during *Izcalli*—the last month of the Mexica calendar.

Ceramic fragments are common in and around the enclosure, and these fragments have been collected by archeologists and dated to the Mesoamerican Classical Period, early Toltec, and Mexica, suggesting that the site was used for ceremonies from the beginning of the Common Era to the collapse of the Mexica Empire in the 16th century (34, 35). Although the constructions have not been dated with precision, early chroniclers reported that the sanctuary in Mount Tlaloc was used by the Toltecs before the 7th century CE (36) and by the Chichimecs in the 12th century, before the arrival of the Aztecs to the Basin (37). It seems likely, then, that the astronomical use and significance of the Mount Tlaloc causeway, and hence the beginning of the Mesoamerican calendar, preceded the founding of Tenochtitlan and the development of the Mexica civilization.

### Solar Alignment with Tepeyac.

Broda (21) noted that the causeway of Mount Tlaloc points toward Mount Tepeyac, a hill that emerges from the Basin’s sediments south of the Sierra de Guadalupe, a range of basaltic mountains in the center of the Basin of Mexico. Indeed, when viewed from Tepeyac, Mount Tlaloc has an azimuth of 100°54′, very close to the bearing of the causeway on Tlaloc’s peak and an elevation of 2°38′ (Fig. 2*A*). Mount Tepeyac (2,280 m) is the southernmost hill of the Sierra de Guadalupe, only 4 km northeast and 7 km east of the pre-Hispanic settlements of Tlatelolco and Azcapotzalco. According to Sahagún (6), the hill had been a place of worship and pilgrimage for the inhabitants of the Basin long before the Spanish Conquest.

Broda’s observation suggests a visual alignment of calendric importance may have existed between the Tepeyac ranges and Mount Tlaloc. Indeed, sunrise alignment with Mount Tlaloc occurs on February 24 (Gregorian date) if viewed from Mount Tepeyac. Like the alignment in the summit’s causeway, the Mount Tepeyac solar alignment date corresponds with that of the causeway and also heralds the beginning of Tena’s new year (Fig. 3*D*). It can be hypothesized, then, that before the Mexica built the Templo Mayor, the inhabitants of the Basin of Mexico were using the alignment between Tepeyac and Mount Tlaloc as a fundamental landmark in their horizon calendar. They could have adjusted with precision their agricultural calendar to the solar year based on the sunrise alignment between Mount Tlaloc and Tepeyac.

### Earlier Alignments of Calendric Significance.

Agriculture was already well established in the Basin of Mexico by the first millennium BCE, largely around the Pre-classic Cuicuilco culture in the southwest of the Basin. The Cuicuilco civilization collapsed in the 3rd century CE when the Xitle volcano became active and covered the whole south of the Basin under a mantle of lava (38). Broda (19) has analyzed the horizon calendar as viewed from the main pyramid of Cuicuilco, built ca. 600 BCE, almost nine centuries before the apogee of the Mexica Empire. She concluded that the sunrise alignment with Mount Papayo (azimuth 89.18° when viewed from Cuicuilco) on March 24, close to the equinox, “could have constituted a simple and effective mechanism to adjust for the true length of the solar year, which needed a correction of 1 d every 4 y.” Broda’s studies on Cuicuilco provide strong evidence suggesting that rigorous calendric calculations and leap-year adjustments were at the heart of the development of Mesoamerican agricultural civilizations from very early times and were certainly very important in pre-Classical settlements. In addition to the equinoctial alignment of sunrise with Mount Papayo, the Cuicuilco observatory would have provided good calendric alignments with Mount Telapon (April 14) and with the “head” of the “sleeping woman” profile of the Iztaccihuatl volcano (February 20). The latter date is very close to Tena’s estimate for the beginning of the Mexica calendric year and, because of Iztaccihuatl’s majestic proportions when viewed from the south of the Basin, could also have constituted an important landmark for calendric adjustments (Fig. 2*C*).

## Discussion and Conclusions

Many early codices seem to validate the working hypothesis that Mount Tlaloc was instrumental in the establishment of the date of the Basin’s new year and in the adjustments necessary to keep the agricultural calendar in synchrony with the solar year. As discussed previously, Sahagún (6) described how *Atlcahualo*, the first month of the year, was devoted to celebrate the *Tlaloc* gods of rain. Similarly, in Duran’s (5) description of the *nemontemi* days, he reported that the year ended when a sign of the first day of the new year became visible above a mountain peak (*SI Appendix*, Fig. S6), suggesting the use of a landmark alignment to indicate the beginning of the new year. Similar associations between Mount Tlaloc and the first day of the new year are shown in other ancient codices, such as Codex Tovar, Codex Borbonicus, and the Wheel of Boban (*SI Appendix*, Figs. S7–S9). The narrow historical relationship between the first month Atlcahualo and Mount Tlaloc has been recently described in detail by Broda (39).

From an ecological perspective, it seems clear that the rugged eastern horizon of the Basin provided precise landmarks that would have allowed to adjust the *xiuhpohualli*, the count of the years, with the true solar calendar. Sahagún’s description of the feasts and ceremonies associated with some of the Mexica “months,” or 20-d periods, coincides well with themes from landmarks visible in the sunrise horizon from the Templo Mayor. Because of its position near the equinox, when viewed from the center of the Basin, Mount Tlaloc seems to have played a very important calendric role. The long causeway at the summit strongly suggests that the ceremonial structure was used as a solar landmark, aligning very precisely with the rising sun on February 23 to 24 and October 19 to 20. The same alignment is found if Mount Tlaloc is viewed from Mount Tepeyac, a holy site whose use as a sacred mount and solar observation post preceded the establishment of the Mexica civilization in the Basin.

These results confirm that, even without the celestial instruments used by Europeans at the time of their arrival (e.g., gnomon, compass, quadrant, and astrolabe), the people in the Basin of Mexico could maintain an extremely precise calendar that would have allowed for leap-year adjustments simply by using systematic observations of sunrise against the eastern mountains of the Basin of Mexico.

## Methods

### Calculation of Azimuths and Elevations.

Coordinates for each site (latitude, longitude, and altitude) were obtained from georeferenced satellite images in Google Earth Pro©. These values were then validated against Mexico's National Institute of Statistics, Geography and Informatics (INEGI) digital topographic charts on a scale of 1:20,000 (https://www.inegi.org.mx/temas/topografia/) and against our own global positioning system (GPS) field readings (*SI Appendix*, Table S1). Altitude estimations were validated by comparing specific points with NASA’s Ice, Cloud, and land Elevation Satellite (ICESat) data on the OpenAltimetry website (https://openaltimetry.org/). Spherical trigonometry formulae (40) were used to calculate haversine distance, azimuthal bearing, and angular elevation between pairs of points given the latitude, longitude, and altitude of each point (*SI Appendix*, Table S2). The calculations were also validated by comparing them with published archeo-astronomic tables by Šprajc (29).

### Astronomical Modeling.

To calculate the position of the sun at any specific day and time, all dates were first converted to Julian Day numbers, a continuous count of days from the beginning of year −4712. The solar coordinates for each specific time were calculated using standard astronomical algorithms (41, 42), including a) the equation of Kepler (eccentric anomaly), b) solar longitude (corrected by the gravitational influence of the moon and planets), c) eccentricity of the Earth’s orbit, d) relative distance to the sun, e) obliquity of the elliptic, f) equation of time, and g) solar declination. Solar angular elevation values were corrected for atmospheric refraction (43), assuming a mean atmospheric pressure of 76 kPa and an air temperature at sunrise of 10 °C. Finally, using spherical trigonometry, solar coordinates were converted to local azimuth and elevation coordinates for a specific site on the Earth’s surface. Simulating the position of the sun every second of the day and knowing the elevation of the landscape horizon above the celestial horizon, the time and azimuth of sunrise were calculated for each day of the year. The calculations were programmed in 64-bit Quick-Basic language (QB64; https://qb64.com/) and compiled as a stand-alone executable program file for faster processing times. The results of our simulations were checked against NOAA’s Solar Position Calculator (https://gml.noaa.gov/grad/solcalc/) to ensure that our results are robust and validate the model.

### Field Validation.

The predictions of the astronomical model were corroborated with field observations. On February 24, 2022, we ascended Mount Tlaloc, camped close to the peak, and climbed to the summit to explore the ancient ceremonial structure. The following day, we ascended the peak once again in the early morning, while still dark, to test the alignment of the rising sun with the stone-walled causeway. We also took ground-level and aerial drone images of the ruins. On the morning of February 26, we visited the sanctuary of Mount Tepeyac at dawn to confirm the alignment of sunrise ca. 1° north of Mount Tlaloc.

## Supplementary Material

Appendix 01 (PDF)

## Data Availability

All study data are included in the article and/or *SI Appendix*.
