# Supplementary material for: Ancient inhabitants of the Basin of Mexico kept an accurate agricultural calendar using sunrise observatories and mountain alignments
Source: Proc Natl Acad Sci U S A. 2022 Dec 12;119(51):e2215615119. doi: 10.1073/pnas.2215615119 (PMC9907100; doi:10.1073/pnas.2215615119)
Supplement: Supplementary file 1 — Appendix 01 (PDF) [file pnas.2215615119.sapp.pdf]

Supporting Information for "Ancient inhabitants of the Basin of Mexico kept an accurate agricultural calendar using sunrise observatories and mountain alignments"

Exequiel Ezcurra[a], Paula Ezcurra[b], Ben Meissner[c]

[a]Department of Botany & Plant Sciences, University of California Riverside, Riverside, California 92521-0147, https://orcid.org/0000-0002-3505-5859, (951) 333 – 5353

[b]Climate Science Alliance, 4640 Cass Street #9220, San Diego, California 92169. https://orcid.org/0000-0002-6545-9133.

[c]Independent filmmaker/photographer, 3916 N Potsdam Ave PMB 4295, Sioux Falls, South Dakota 57104.

Supporting Information Tables

Table S1. Geographic coordinates (latitude and longitude in decimal degrees, altitude in meters) of the three main astronomical observation sites and seven conspicuous landmarks on the eastern horizon in the Basin of Mexico.

Solar observatories in the Basin of Mexico

Site	Latitude	Longitude	Altitude (m)
Tepeyac	19.4868	-99.1157	2,280
Templo Mayor	19.4349	-99.1314	2,232
Cuicuilco	19.3016	-99.1816	2,301

Eastern horizon landmarks in the Basin of Mexico

Site	Latitude	Longitude	Altitude (m)
Tlamacas	19.5447	-98.7055	3,158
Monte Tláloc	19.4123	-98.7125	4,122
Telapón	19.3709	-98.7198	4,058
Papayo	19.3076	-98.6989	3,652
Iztaccihuatl (head)	19.1869	-98.6459	5,064
Iztaccihuatl (peak)	19.1784	-98.6421	5,205
Popocatepetl	19.0227	-98.6279	5,393

Table S2. Azimuthal bearings and angular elevations of seven conspicuous landmarks on the Basin's eastern horizon viewed from the three main astronomical observation sites in the Basin of Mexico.

Horizon landmarks	Observatories		
	**Tepeyac**	**Templo Mayor**	**Cuicuilco**
	**Azimuthal bearings (degrees)**		
Tlamacas	81.43	74.64	61.50
Monte_Tlaloc	101.03	93.21	75.89
Telapon	107.19	99.30	80.90
Papayo	114.44	107.26	89.17
Iztaccihuatl (head)	124.00	118.35	102.69
Iztaccihuatl (peak)	124.53	118.97	103.51
Popocatepetl	135.14	130.84	117.98
	**Angular elevation (degrees)**		
Tlamacas	0.96	0.93	0.61
Monte_Tlaloc	2.25	2.26	1.82
Telapon	2.14	2.19	1.83
Papayo	1.42	1.49	1.30
Iztaccihuatl_head	2.40	2.53	2.48
Iztaccihuatl_peak	2.50	2.63	2.58
Popocatepetl	2.12	2.27	2.38

Supporting Information Figures

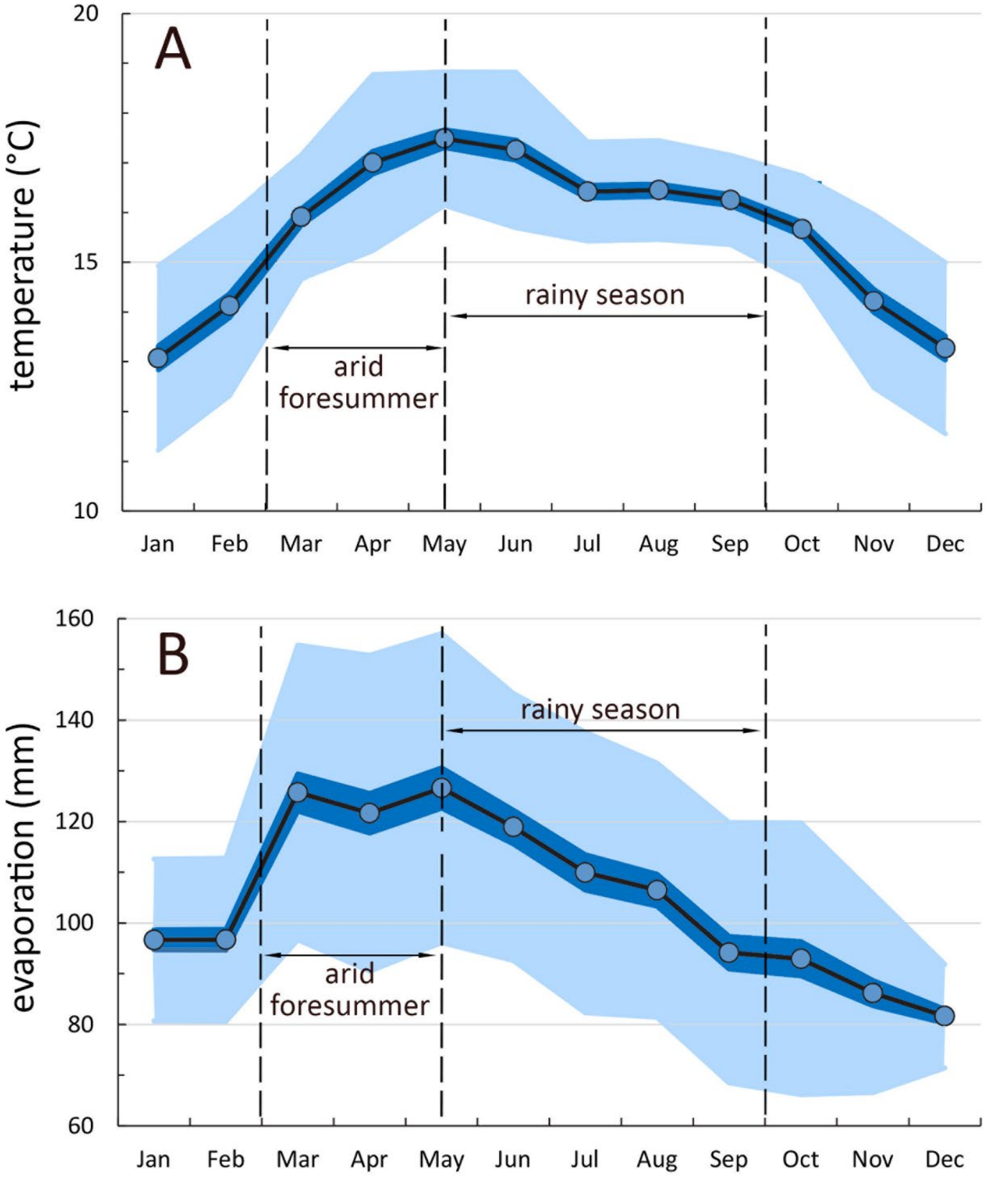

Figure S1. (A) Mean monthly temperatures in the Basin of Mexico (blue points; blue shaded area: ±1 standard error; light blue area: ±1 standard deviation). (B) Mean monthly cumulative evaporation, a proxy measure of evaporative demand for plants. Note that the highest temperatures and evaporative demands occur during the dry spring months, from March to mid-May (data from years 1952–2016, Weather station 9020 Pedregal, *Comisión Nacional del Agua*, Mexico).

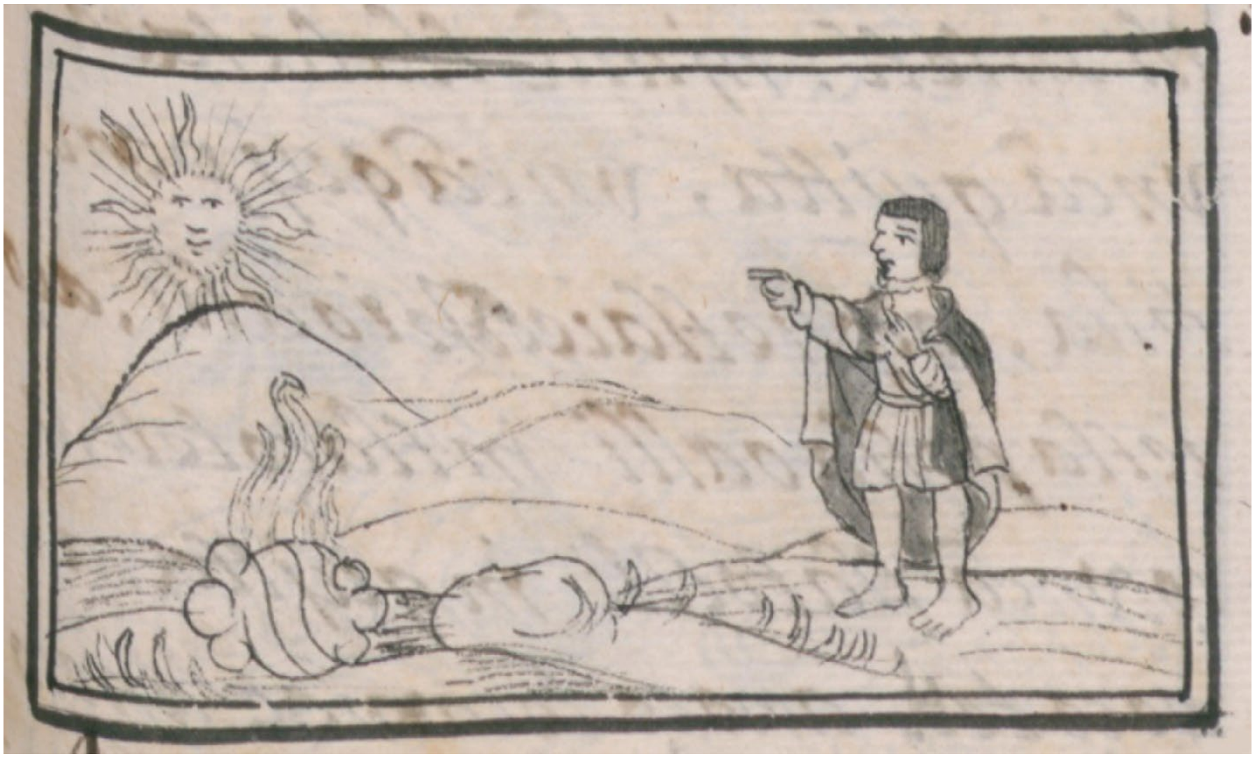

Figure S2. A native expert in mineral ores uses bearings of the rising sun against mountains in the horizon to relocate deposits of precious minerals (Fray Bernardino de Sahagún, 1575, *The Florentine Codex*, digital facsimile available at https://www.loc.gov/item/2021667856).

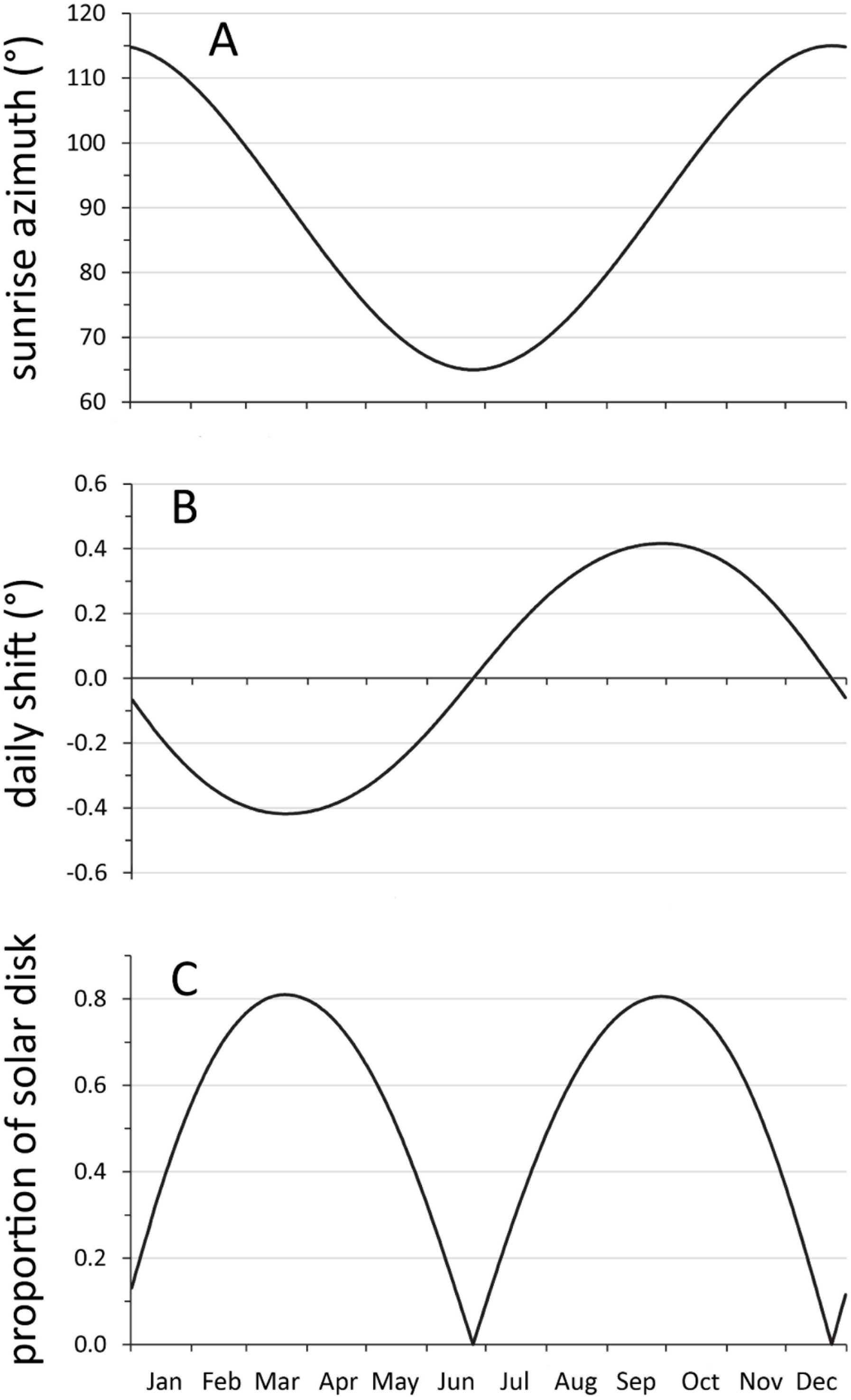

Figure S3. (A) Azimuth of the rising sun against the celestial horizon at the latitude of the Basin of Mexico (19°26’). (B) Daily shifts in sunrise azimuth in the Basin of Mexico from one day to the next, expressed in sexagesimal degrees. (C) Daily shifts in sunrise azimuth in the Basin of Mexico from one day to the next, expressed as a fraction of the solar disk (absolute values).

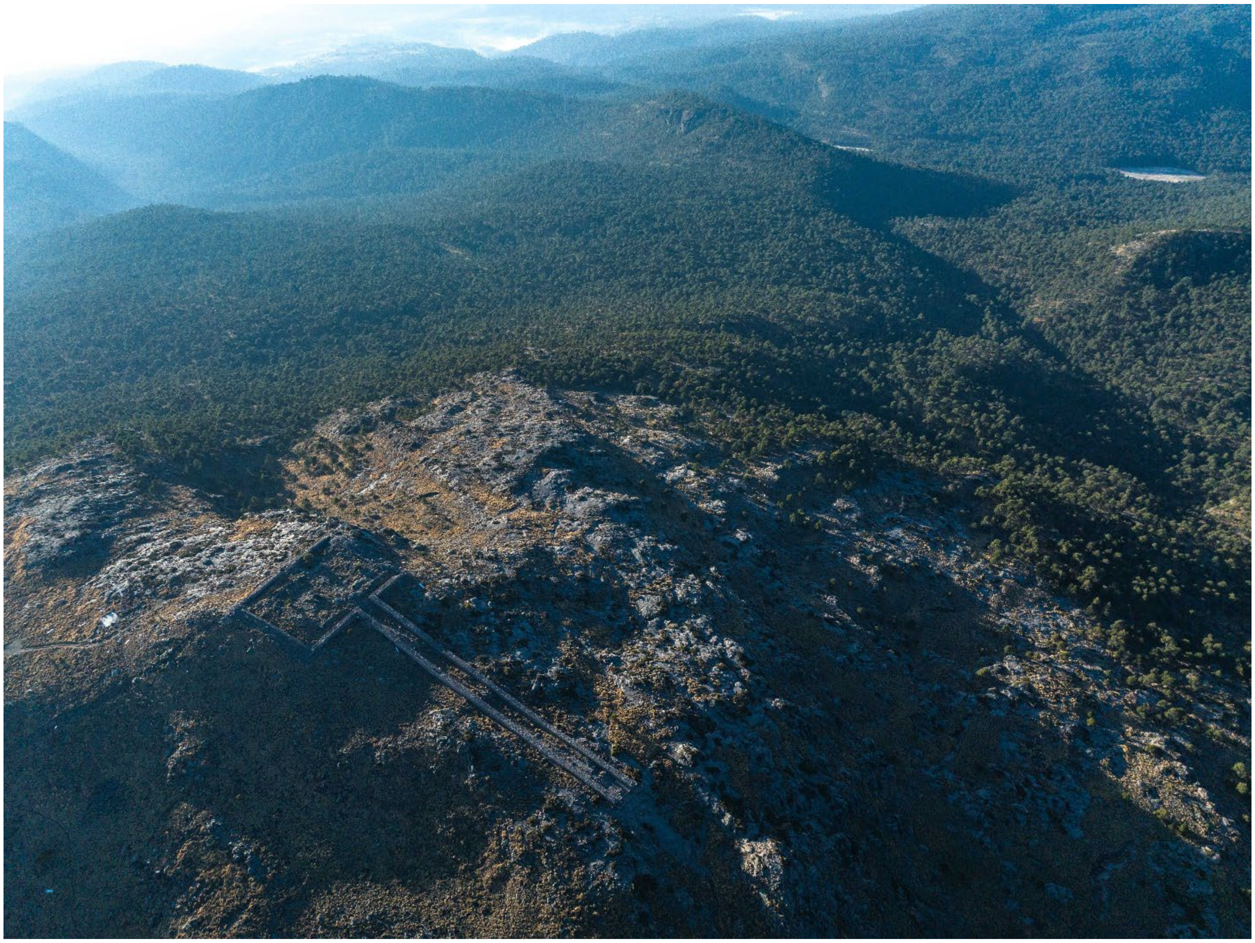

Figure S4.The ceremonial compound at Mount Tlaloc, photographed from a drone on the morning of February 25, 2022. Photo credit: Ben Meissner.

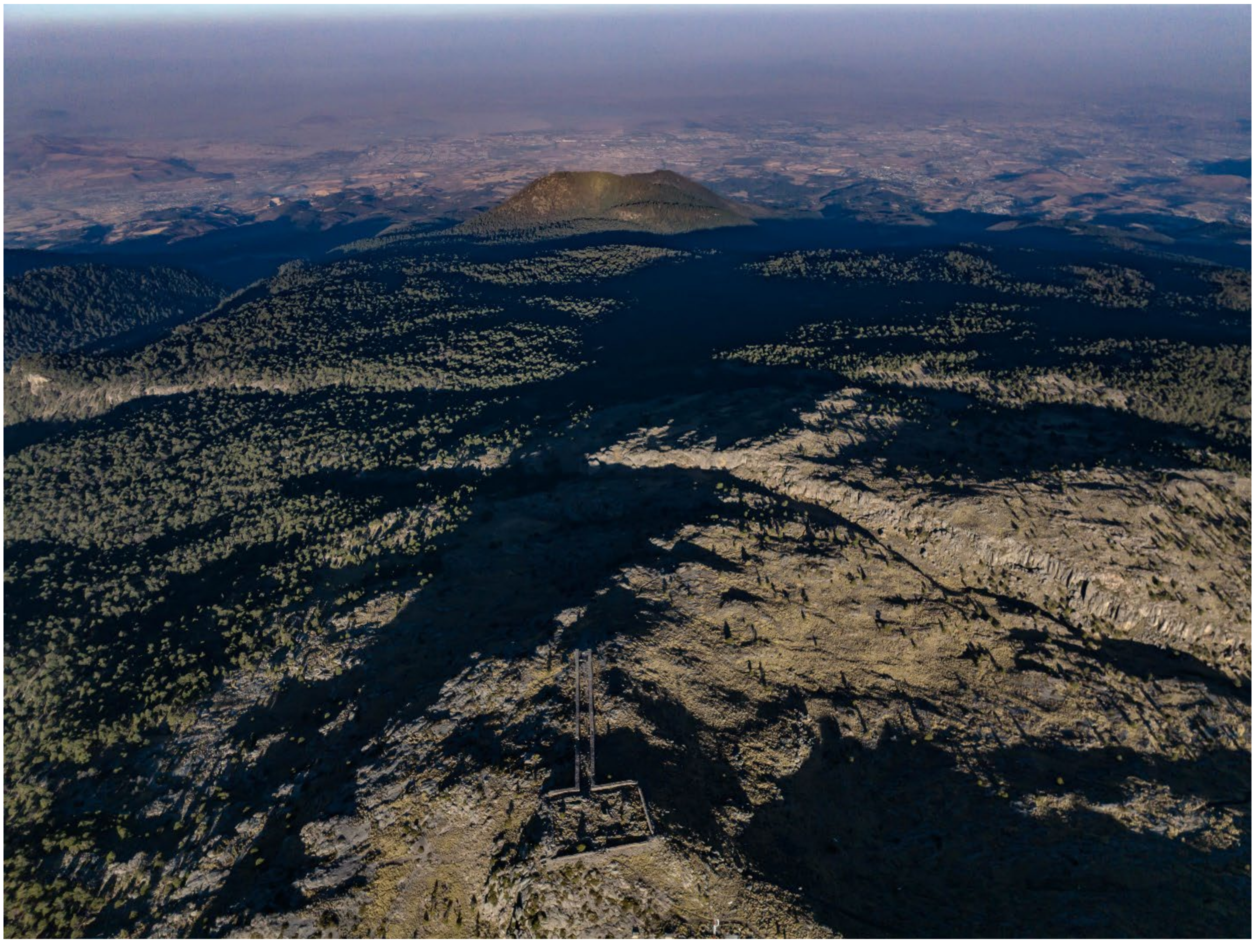

Figure S5.The ceremonial compound at Mount Tlaloc, photographed from a drone on the morning of February 25, 2022. Note the alignment of the causeway with the shadow of the trees. In the distance, barely visible in the smog of Mexico City, the Sierra de Guadalupe and Mount Tepeyac.
Photo credit: Ben Meissner.

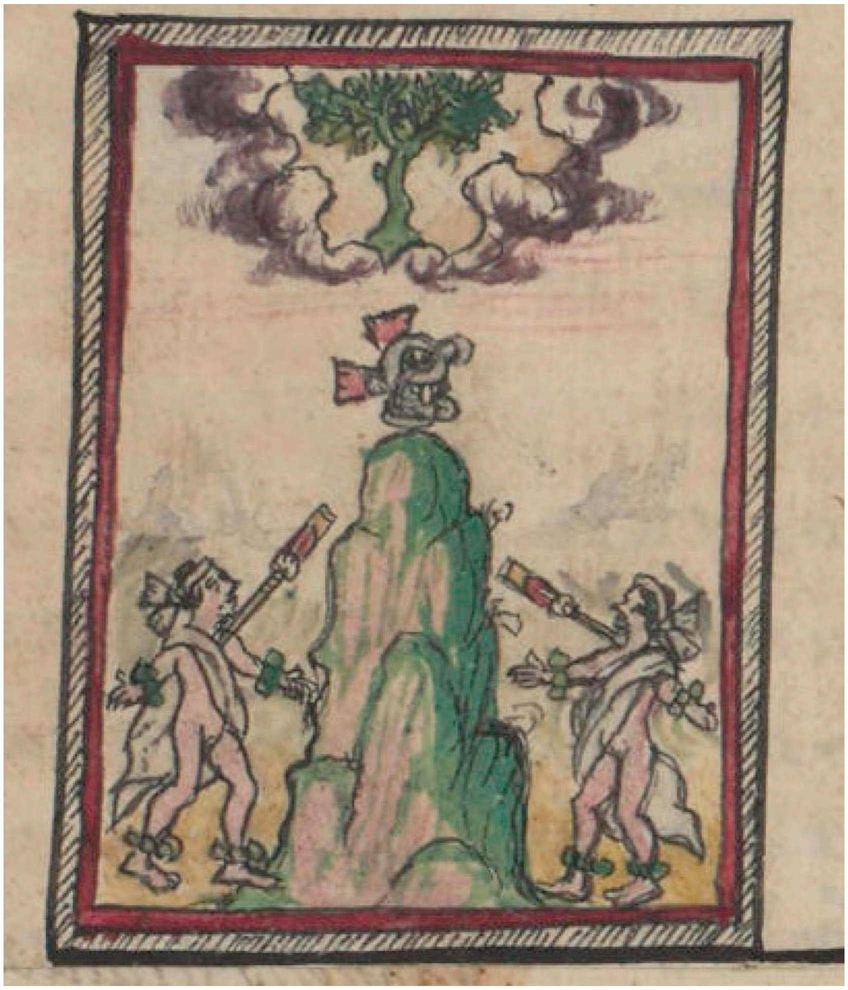

Figure S6. End-of-year illustration from Codex Durán (1). The *nemontemi* or useless days at the close of the year end when a sign of the first day of the year (represented in the illustration by the symbol *cipactli*, or serpent's head) becomes visible above a mountain peak. Also aligned with the mount, a tree is represented appearing in the clouds, suggesting some type of celestial correlation with the end of the year. The allegory of a landmark alignment is strikingly apparent. The text, in ancient Castillian, reads: "We all know well that the year has 365 days; these indians counted only 360 and called the five remaining days excess, or useless days, and did not give them names like the other days, nor figures; leaving them blank like ill-omened days. They called them *nemontemi*, that means without need or benefit. During those five days they fasted and made great penance of abstinence. They did not eat but once a day, and that meal was only of dry tortillas. They would self-flagellate, bleed, and withdraw from their women. They considered those that were born during these days persons of bad luck. They observed in this period their leap years, in the same manner as we do, and if we note the figure in the illustration, we will see that above a mount a symbol is shown that indicated the beginning of the month, and although this period ended in the rose symbol they had this other symbol together with it, to transition the rose on to the serpent's head" (authors' translation, see endnote 1 for original text in Spanish).

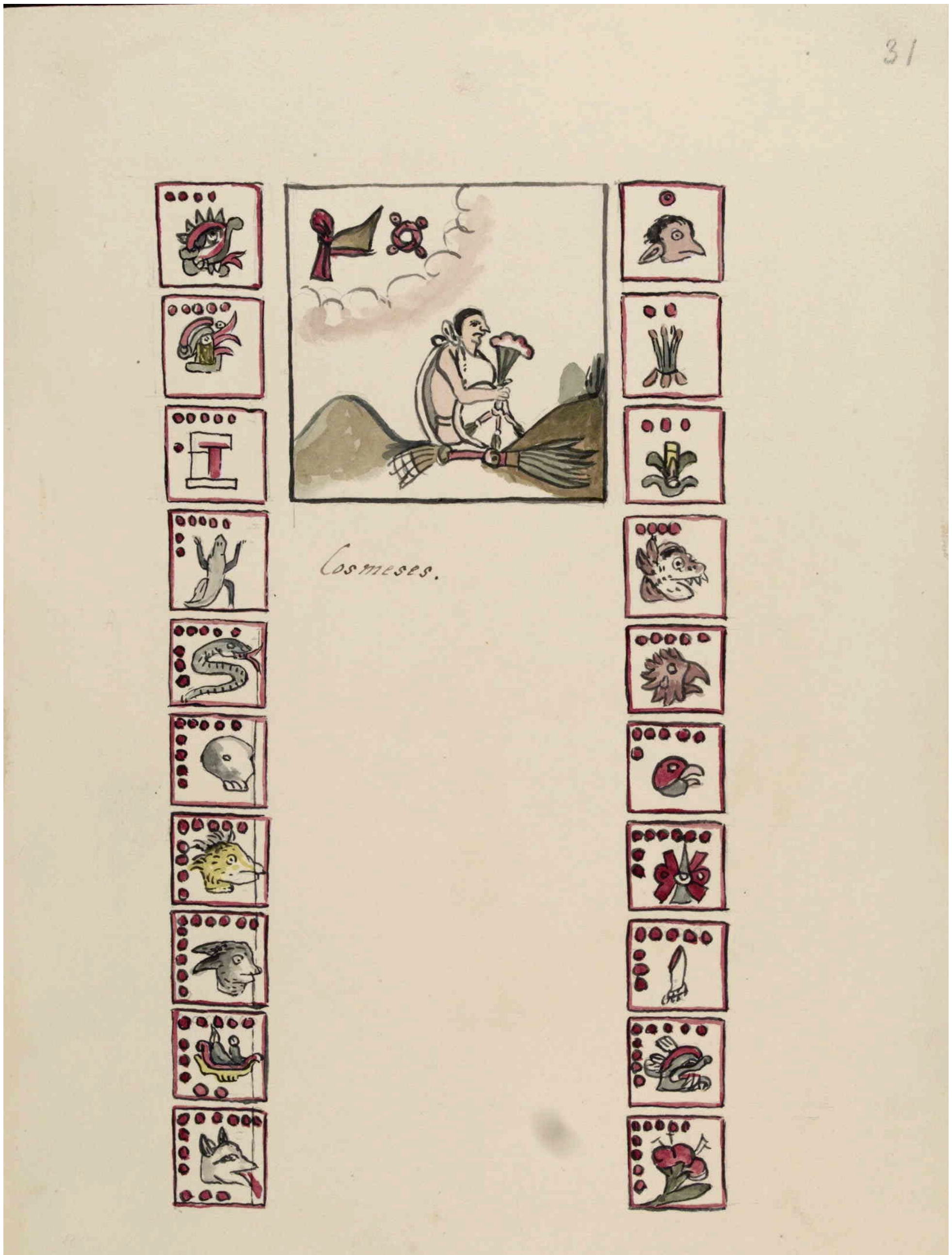

Figure S7. Representation of the first month of the year in the Tovar Codex. According to Durán, the names for the first month were *Atlcahualo* (water ends), *Cuauhtlehua* (trees sprout), and *Xochtzitzquilo* (gathering of flowers). All three are related to the beginning of Mesoamerica's arid pre-monsoon, which starts in late February (see Fig. 1 and main text). At this time of the year aridity increases, tropical trees flower before the rains set in, it is indeed the time to collect some of the last winter flowers before everything dries up with the rising spring temperatures. In the foreground of the image, a young man is seen gathering flowers. A mountain is seen in the background with two symbols appearing in the sky: a *tetehuitl*, or festival banner, and *ilhuitl*, a festival day (2). Note the allegory of a mountain alignment with celestial bearings as a symbol of the Basin of Mexico's new year.
Source: Library of Congress Rare Book and Special Collections, Division Jay I. Kislak Collection, Washington, D.C. 20540; http://hdl.loc.gov/loc.rbc/kislak.81897.1

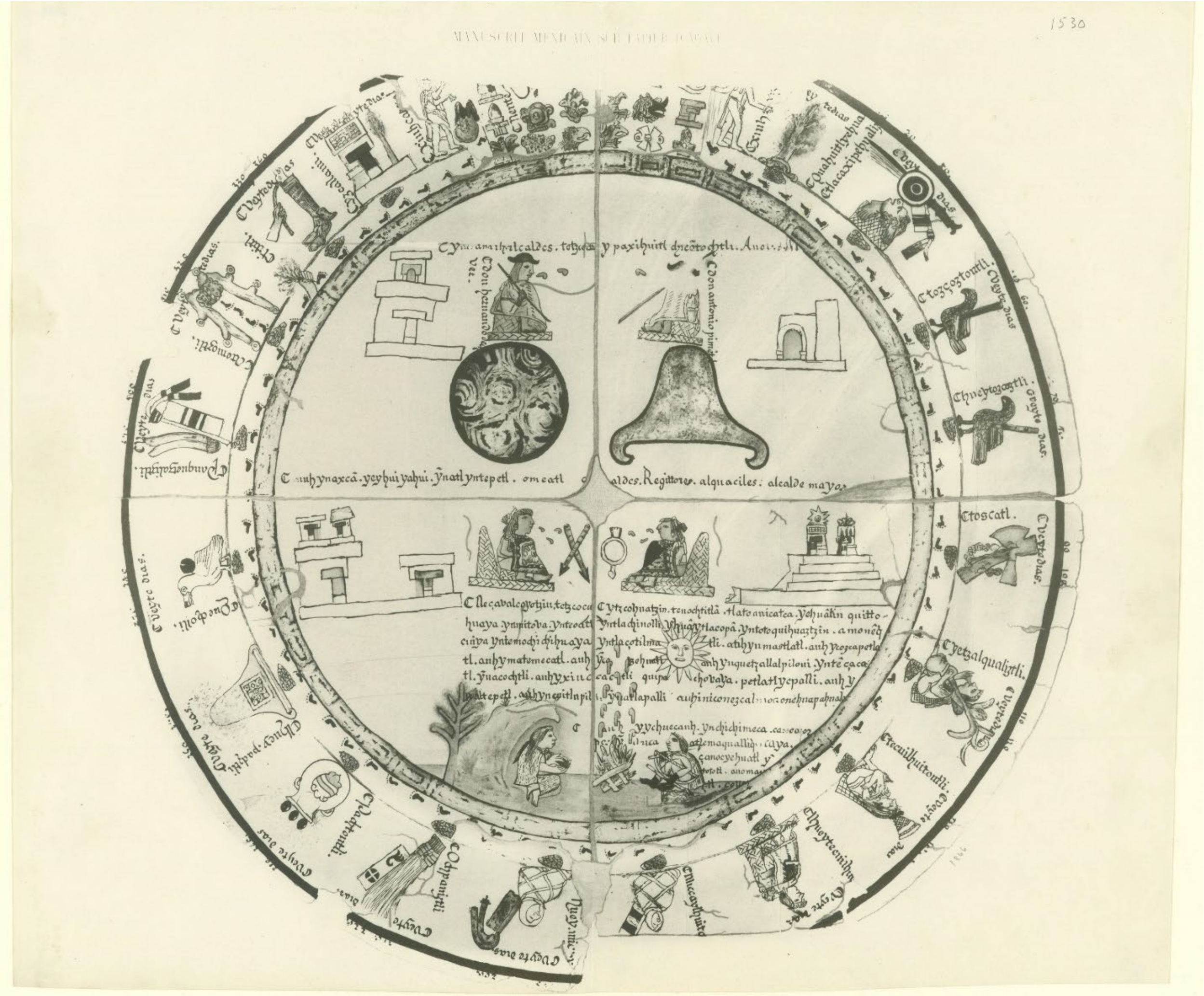

Figure S8. The Boban Calendar Wheel, made towards the end of the 16th century, shows the 18 Mexica "months" with the five *nemontemi* days on top (3). A sixth day, represented as two corn cobs on a clay pot is seen to the left of the five nemontemi at the side of a *tecpatl* year, indicating that an additional *nemontemi* was added every four years (4). The two symbols of Texcoco, the lake and Mount Tlaloc, are seen in the upper two interior quadrants, with the two colonial rulers (*tlatoani*) of Texcoco: Antonio Pimentel Tlahuitoltzin (1540–1545) and Hernando Pimentel Ihuian (1545–1564). Although at the time this codex was made the Spanish Inquisition had banned the worshiping of Aztec gods, Mount Tlaloc still is shown as associated to the calendric new year (5).
©John Carter Brown Library, Brown University, Providence, R.I. 02912; downloaded from https://jcb.lunaimaging.com/luna/servlet/detail/JCB~1~1~1035~1190002

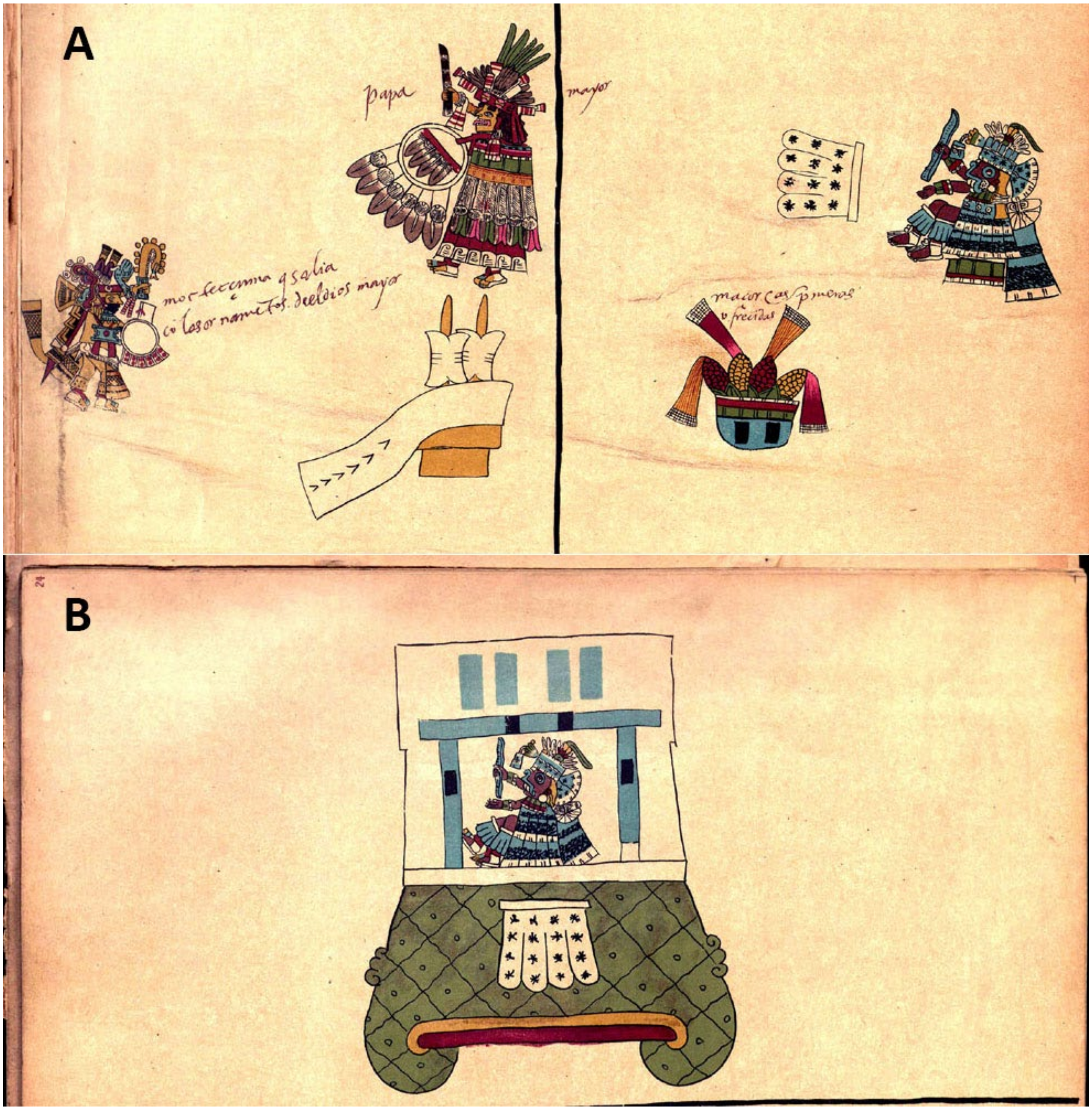

Figure S9. Tlaloc in the Codex Borbonicus. (A) This image depicts the transition from *Izcalli*, the last month of the year (left) to *Atlcahualo*, the first month of the new year. Over an altar decorated with paper, Moctezuma dressed as *Xuihtecuhtli*, the god of fire, and a high priest dressed as Cihuacóatl, the woman-serpent (left), present a new-year offering of corn to Tlaloc (right). (B) In other parts of the Codex Borbonicus Tlaloc is represented as a living in his temple on top of the mountain. See Anders, Jansen, and Reyes-García (6) for details on the interpretation of the Codex Borbonicus. Images downloaded from: Foundation for the Advancement of Mesoamerican Studies, Inc.; http://www.famsi.org/research/loubat/Borbonicus/

End notes

1. Original text for Figure S5 (excerpt from Durán; “Días demasiados”, p. 305): “*Bien sabemos todos como el año tiene trescientos y sesenta y cinco días estos indios contaban los trescientos y sesenta y á los cinco dias que había llamaban los dias demasiados y sin necesidad y así no les daban nombres como á los demas ni figuras y así los dejaban en blanco y como á dias aciagos les llamaban nemontemí que quiere decir dias sin necesidad ni provecho. Estos cinco dias ayunaban y hacían grandes penitencias de austinencias de pan y agua no comían mas de una vez al dia y esa comida era de tortillas secas azotabanse sangrábanse apartábanse de sus mujeres tenían por de mala suerte á los que en estos dias nacían hacían en este mes su bisiesto de la mesma manera que nosotros le hacemos y si notamos la figura de la pintura veremos que encima de un cerrillo está pintada la letra dominical que á ellos les era principio de mes y aunque este dia acababa en el signo de rosa tenían estotra juntamente con ella para mudar la rosa en la cabeza de sierpe*.”

2. The *Xiuhpohualli* was a 365-day calendar used in pre-Columbian times by Nahua peoples in central Mexico. It was composed of eighteen 20-day "months" with a separate 5-day period at the end of the year called the *nemontemi*, or “useless,” days. The name *Xiuhpohualli* means “the count of the seasons” in the Nahuatl language. It is formed by the junction of two words: *xihuitl* and *pohualli*. The latter, *pohualli*, is the noun for a count. The former, *xihuitl*, has two different meanings: On the one hand, *xihuitl* or *xiuh*, is the noun used to describe seasonal herbs. On the other hand, *xihuitl* is also the name of the solar year (7). This double meaning seems to support a main hypothesis behind our study: The ecological cycle of seasonal herbs was seen as an indicator of the agricultural cycle related to the solar year.

References

1. D. Durán, *Historia de las Indias de Nueva España y Islas de Tierra Firme* (printed edition of the 16th century codex, Imprenta de Ignacio Escalante, México, 1880). 305 pp.

2. M. Thouvenot, *Ilhuitl* (día, parte diurna, veintena) y sus divisiones. *Estudios de Cultura Náhuatl* 49: 93–160 (2015).

3. C.E. Dibble, The Boban calendar wheel. *Estudios de Cultura Náhuatl* 20: 173–182 (1990).

4. R. Tena, Rafael, El calendario mesoamericano. *Arqueología Mexicana* **41**, 4–11 (2000).

5. B. Benton, *The Lords of Tetzcoco: The Transformation of Indigenous Rule in Postconquest Central Mexico* (Cambridge University Press, New York, 2017) 212 pp.

6. F. Anders, J. Maarten, L. Reyes-García, *El Libro del Ciualcóatl: Homenaje para el Año del Fuego Nuevo (Libro explicativo del llamado Códice Borbónico)* (Fondo de Cultura Económica, México, 1991) 251 pp.

7. S. Wood (ed.), *Online Nahuatl Dictionary* (Wired Humanities Projects, College of Education, University of Oregon, Eugene, Oregon, 2000). Consulted at https://nahuatl.uoregon.edu/
